# Heart rate sensor validation and seasonal and diurnal variation of body temperature and heart rate in domestic sheep

**DOI:** 10.1016/j.vas.2019.100075

**Published:** 2019-10-01

**Authors:** Boris Fuchs, Kristin Marie Sørheim, Matteo Chincarini, Emma Brunberg, Solveig Marie Stubsjøen, Kjell Bratbergsengen, Svein Olav Hvasshovd, Barbara Zimmermann, Unni Støbet Lande, Lise Grøva

**Affiliations:** a Inland Norway University of Applied Sciences, Faculty of Applied Ecology, Agricultural Sciences and Biotechnology, NO-2418 Elverum, Norway; b Norwegian Centre for Organic Agriculture (NORSØK), Gunnars veg 6, N-6630 Tingvoll, Norway; c University of Teramo, Faculty of Veterinary Medicine, S.P 18, Loc. Piano d'Accio, 64100 Teramo, Italy; d Norwegian Veterinary Institute, Department of Animal Health and Food Safety, P.O. Box 750 Sentrum, N-0106 Oslo, Norway; e Norwegian University of Science and Technology (NTNU), Department of Computer Science NO-7491 Trondheim, Norway; f Norwegian Institute of Bioeconomy Research (NIBIO), Division of Forestry and Forest Resources, Gunnars veg 6, NO-6630 Tingvoll, Norway

**Keywords:** Heart rate, Sheep, Sensor, Bio logging, Body temperature

## Abstract

Advantages of low input livestock production on large pastures, including animal welfare, biodiversity and low production costs are challenged by losses due to undetected disease, accidents and predation. Precision livestock farming (PLF) enables remote monitoring on individual level with potential for predictive warning. Body temperature (Tb) and heart rate (HR) could be used for early detection of diseases, stress or death. We tested physiological sensors in free-grazing Norwegian white sheep in Norway. Forty Tb sensors and thirty HR sensors were surgically implanted in 40 lambs and 10 ewes. Eight (27%) of the HR and eight (20%) of the Tb sensors were lost during the study period. Two Tb sensors migrated from the abdominal cavity in to the digestive system. ECG based validation of the HR sensors revealed a measurement error of 0.2 bpm (SD 5.2 bpm) and correct measurement quality was assigned in 90% of the measurements. Maximum and minimum HR confirmed by ECG was 197 bpm and 68 bpm respectively. Mean passive HR was 90 bpm (SD = 13 bpm) for ewes and 112 bpm (SD = 13 bpm) for lambs. Mean Tb for all animals was 39.6°C (range 36.9 to 41.8°C). Tb displayed 24-hour circadian rhythms during 80.7 % but HR only during 41.0 % of the studied period. We established baseline values and conclude that these sensors deliver good quality. For a wide agricultural use, the sensor implantation method has to be further developed and real-time communication technology added.

## Highlights

• The heart rate sensors measured reliable heart rates and assigned the quality correctly
• Free grazing sheep had passive heart rates of 90 bpm to 112 bpm depending on age
• Body temperature followed a convex curve peaking in summer
• Heart rate followed a concave curve peaking in summer
• All sheep's body temperature displayed 24-hour circadian rhythms

## 1 Introduction

Low input animal production on range pastures is considered to provide great behavioral freedom to the animals and a high level of animal welfare. Animals on range pastures do however receive less frequent attention than animals on fenced pastures, and farmers therefore detect and treat disease and injuries at a later stage (Dwyer, 2008). In order to ensure animal welfare and production when grazing these pastures, there is a need for increased surveillance of health states and behavior on individual and flock level. Implementing new technology into rangeland grazing systems has potential to assist farmers in reaching these goals.

Societies increasingly demand sustainable, high animal welfare standard food products (Reganold and Wachter, 2016) and especially in European countries, believe in individual attention for the animals by the farmers (Verbeke and Viaene, 1999). In contrast to this, the trend of increased productivity and farm size leads to higher number of animals per stockperson and farmers having less time to physically observe single animals (Hume et al., 2011). The farmer dealing with increasing stock size and efficiency while keeping control over health, welfare and economics, needs assistance from modern technology. Precision livestock farming (PLF) is continuous, automated real time monitoring on individual level and an important tool and contributor to the next technology revolution in agriculture (Van Hertem et al., 2017). In order to increase productivity, reducing losses due to disease and stress will be necessary (Hume et al., 2011). Implementing sensors in farming systems enables the farmer to measure needs and performance of his stock in order to fine-tune growth rates and early detection of potential problems. Various products have been launched ranging from acoustic surveillance of swine cough (Guarino et al., 2008), the monitoring of ruminating and eating activity in dairy cows (Zehner et al., 2017), using image analysis to asses movement behavior in poultry (De Wet et al., 2003) or WI-FI enabled radio frequency identification (RFID) chips to identify ownership of semi domestic reindeer (Mustafa, 2015).

In Norway, vast rangeland resources are available for sustainable food production and more than 2 million ewes and lambs graze on unfenced rangeland pastures during the summer months every year (NIBIO, 2017a). This behavioral freedom brings both high animal welfare qualities, optimized foraging and high weight gains. However, estimated losses of about 10 million euros per year due to disease, accidents and predation are compromising these advantages. Since 2010, between 5% and 7% of all lambs were lost per year (NIBIO, 2017a), ranging above 30% in some grazing areas (NIBIO, 2017b). Causes of losses are difficult to determine, as dead sheep usually remain undetected. In 2016, farmers reported a total loss of 75,272 sheep on range pastures and received compensation for 17,794 of those. However, less than 3000 sheep had a documented cause of death (NIBIO, 2017a; Rovbase, 2018).

The physiological state of wild and domestic animals is a key factor for performance and production. Technology development in bio-sensors enables measurement of a set of physiological traits, helping to assess the allostatic load and possibly define its stressors (Wikelski and Cooke, 2006). Heart rate (HR) and body temperature (T_b_) have been used to detect disease (Marais et al., 2013), stress (Evans et al., 2016), gestation and parturition (Friebe et al., 2014; Trethowan et al., 2016; Thiel et al., 2019) and estrus (Andersson et al., 2016) in free ranging terrestrial mammals and production animals. In many species, oxygen consumption and HR are closely related and although calibration is needed, HR can be used as a proxy for metabolic rate (Green, 2011). Metabolic rate relates to food consumption and efficiency and can be influenced by seasonal, environmental and physiological factors (Baldock and Sibly, 1990; Baldock et al., 1988; Nkrumah et al., 2006). Shearing of domestic sheep leads to a sudden change of heat exchange and requires adjustment of the feed or ambient temperature regime (Piccione et al., 2002). In endothermic animals T_b_ can be used to detect fever which is closely related to infectious diseases and based on a long evolutionary history (Ewald, 1980).

The development in computer storage technology and battery capacity provides implantable sensors measuring HR and T_b_ with the potential for logging the entire production cycle of the animals. Costs of reliable systems have been high and so far predominantly used in research settings, mainly on marine animals and birds (Fehlmann and King, 2016). Future development will likely decrease costs of physiological sensors and make them available for animal producers. In livestock production, automated early detection of estrus, parturition, disease, optimized metabolic rate and behavior can be of high economical value and have a significant impact on animal welfare.

In this study we used domestic sheep that freely range on unfenced mountain pastures during summer in Norway to develop and validate the technique of continuous HR and T_b_ logging in livestock production. Our goal is to elaborate standard values that can be used as comparison for alternative ways of real-time logging T_b_ and HR in sheep. Implantation of relatively small and light devices can be preferable over external devices if implantation is done by trained personnel and wound healing likely to be undisturbed by the animals’ behavior and ecology (Forin-Wiart et al., 2018). For validation purposes, we used implantable systems in order to get high quality continuous measure of seasonal core T_b_ and HR in free ranging sheep. The aims of this study were: a) to establish baseline values, diurnal and seasonal trends and demographic variation in T_b_ and HR for domestic free-ranging sheep, b) to validate the quality of the HR measurements with ECG, and c) to estimate the impact of the implantation process and the application of a sensor on the growth performance of sheep during the grazing season.

## 2 Material & Methods

### 2.1 Study herds

We selected Norwegian white sheep (NWS) from two different sheep herds. The coastal herd was located in Tingvoll municipality in western Norway (62.9861 N, 8.2482 E), an area with known high incidence of tick-borne fever (TBF) (Grøva et al., 2013). The inland herd was located in an inland mountain area in Tynset municipality in central Norway (62.3169 N, 10.9534 E). Ticks are not prevalent in this area (Jore et al., 2011) but losses to predators such as wolverine *(Gulo gulo)*, brown bear *(Ursus arctos)*, lynx *(Lynx lynx)* and wolves *(Cannis lupus)* are frequent*.* In the coastal area, average summer temperature (June-August) is 12.7°C and precipitation averages 1160 mm per year (NRK, 2017). The coastal pasture ranges from 140 to 650 meters above sea, covers about 4000 ha and 1,500 sheep released in 2016. The inland climate is continental, with average summer temperature (June – August) of 11.7°C and a yearly precipitation of 440 mm (NRK, 2017). The inland pasture ranges from 480 to 980 meters above sea level, covers approximately 4500 ha and 2100 sheep were released in 2016.

### 2.2 Study animals

We selected 10 ewes with twin lambs (n=20) from each of the two herds. Ewes were selected for similar lambing dates and an equal sex ratio among lambs. We implanted a temperature T_b_ sensor (Centi-T version 14, Star Oddi, Gardabaer Iceland) in one of the twin lambs and a HR sensor (Milli-HRT version 8, Star Oddi, Gardabaer Iceland) and a T_b_ sensor in the second lamb. In the inland herd, we also implanted HR sensors in the 10 ewes (mothers). In the coastal herd mean age of the lambs at implantation was 49 days (range from 45 to 56 days), and the sex distribution was 11 females and 9 males. In the inland herd mean age of the lambs at implantation was 51 days (range from 46 to 57), and sex distribution was 8 females and 12 males. In both herds, we assigned control groups consisting of the rest of the flock of the same breed; i.e. 35 lambs in the coastal herd and 195 lambs in the inland herd. All study animals were released on range pasture.

Sensors were retrieved at slaughter and data was downloaded from the sensors post retrieval using a communication box and the Mercury software v4.50 (Star Oddi, Gardabaer Iceland). Mean age of lambs at slaughter from the coastal herd (including the control lambs) was 136 days (range from 119 to 152 days). In the inland herd, sensors were removed at slaughter from all lambs and six of the ewes at a large industrial slaughter factory. Sensors were retrieved from the carcasses within the slaughter line. Three ewes were kept for breeding and the sensors were surgically removed. Mean age of all lambs at slaughter from the inland herd was 144 days (range from 125 to 162 days).

### 2.3 Surgical implantation

The surgery was conducted on the basis of veterinary medical principles and methods. For each surgery a protocol was written. The animals got analgesic medication after the surgery and postoperative follow up. All sensors were sterilized using a 12-hour gas sterilizer and propylene gas. For more details, see appendix.

### 2.4 Ethical permit statement

All experiments were approved by the Norwegian Food Safety Authority (FOTS ID 8561) and followed the ARRIVE guidelines (Kilkenny et al., 2010).

### 2.5 Post-operative follow-up

The lambs of the coastal herd stayed in the barn for two days and then fenced on a nearby pasture for four days post-surgery and they were clinically examined morning and evening. Six days after the surgery, all lambs were collected and clinically examined and rectal temperature measured. Lambs with signs of TBF (rectal T_b_ > 40.5°C or/and clinical signs of a local tick borne disease) were treated with antibiotics (Terramycin prolongatum vet ®; ATCvet-nr.: QJ01A A06). The same procedure was followed at day 15 post surgery. After that, the lambs were observed morning and evening every day until they moved to the summer range pasture and observed 2-3 times every week, until they were collected from the range pasture and brought home at the end of August.

The inland herd was kept on a pasture beside the farm for one week post implantation and then transported to the summer range pasture. Post-operative follow up was less intense compared to the coastal herd, but all animals were observed twice per day during the first week. They were collected from summer range pastures and transported back to the farm during the end of August.

### 2.6 Programming of HR sensors

The HR sensor has a single channel three electrode ECG amplifier that measures electrical activity in microvolts (mV). The measurement frequency was set on 200 Hz for 3 seconds. In order to save memory, a real-time ECG signal processing algorithm transfers the raw data in to a HR in beats per minute (bpm) and a quality assignment of the measurement ranging from 0 to 3 (3 as lowest). After reviewing the recorded data, Star Oddi suggested a threshold < 2 for the algorithm measurement quality. The quality assignment is based on the regularity of the R-R intervals per measurements; i.e. if an R peak is not recognized, the interval doubles in length, and the algorithm controls for the missing peak and lowers the quality assignment, more details about the function can be found in Bjarnason et al. (2019). We programed three different sensing intervals: 1) A basic interval sensing a HR every 10 min; 2) For behavioral studies, we programed three periods with HR logging every 2 minutes for one week each; and 3) For evaluation purposes, we configured the sensors to store HR values every minute during a 20 minute period. Four evaluation periods, spread equally over the entire deployment period were programed. During the evaluation sequence raw data was stored, enabling an ECG based manual evaluation of the on board process. We did not further analyze the subcutaneous T_b_ measured by the HR sensor.

### 2.7 Programming of T_b_ sensors

The T_b_ sensors were programed to record temperature every minute during the entire study period. Every sensor is delivered with a calibration certificate for the thermometer accuracy over a range from 0° to 45°C. The Centi-T sensors used in this study had a factory reported average mean measurement error of 0.01°C or lower and the Milli-HRT sensors of 0.025°C or lower.

### 2.8 HR evaluation process

For each measurement during the evaluation period, the raw ECG were plotted on a standard ECG strip. First, a visual assessment of the signal quality was performed and each ECG was classified into four classes (manual quality, here written as roman numerals I to IV). Class I: QRS complex clearly visible; amplitude of the measurement regular; no or very little noise. Class II: QRS complex visible; amplitude of the measurement regular; P, T and U waves partly covered by noise. Class III: R or S peaks visible, amplitude irregular; noise covers details. Class IV: Noise makes interpretation impossible. We compared this manual quality assessment with the quality assignment of the algorithm. Of particular interest were false real or real false. False real were considered if the algorithm-assigned quality was below 2 but the manual assignment was of class IV, detecting a HR from noise. Real false were defined as a quality assigned by the algorithm above 2 and a manual assignment of II or better, removing measurements of good ECG quality.

In a next step, 600 ECG records of manual quality I – III and an algorithm-assigned quality of 0 were randomly selected. For each ECG strip, the first and last RR interval was measured and a HR in bpm was calculated from the mean RR interval length. The sensor-provided HR was compared to the manually measured HR. We used a linear mixed model (Bates et al., 2015) with HR provided by the sensor as the response variable and the manually measured HR along with the evaluation period and age class as fixed factors and the animal ID and evaluation period as random slopes. We first selected a random structure based on AIC followed by a stepwise backward selection based on P-values of the fixed factors (Zuur et al., 2009). All intercepts were forced to zero. All data processing, plotting and analyzing was performed using R (Versions 3.3.1 – 3.4.2) (R Core Team, 2014).

### 2.9 Mean heart rate

Mean heart rate was calculated for active and inactive behaviors. Six randomly selected family groups (ewe and two lambs) were approached based on GPS positions on the mountain pasture and observed for at least 20 minutes in either active or inactive behavior. We noted the position of the sheep and its behavior in parallel according to an a priori defined ethogram. The positions were later grouped into “active” and “passive” for analysis. “Active” was considered if the sheep was standing, walking or running, “passive” if the sheep was laying down for ruminating, resting or sleeping. Mean heart rates were calculated for both sex and age groups. During the quality assessment of the ECG strip, we selected measurement candidates for calculation of maximum and minimum HR. On those measurements, we manually measured all R-R intervals to calculate HR.

To analyze seasonal patterns of HR in domestic sheep we fitted a generalized additive mixed model (function bam) using the R packages mgcv (Wood, 2011) and itsadug (van Rij et al., 2016) with the daily mean HR per individual as the response. After filtering for quality 0 & 1, the number of measurements per hour of the day ranged between n=6880 (06:00 to 06:59) to n=7560 (23:00 to 23:59). We decided not to account for uneven sampling throughout the day when calculating the daily mean. All HR measurements three days post implantation and the days of slaughtering were excluded from the analysis. As fixed predictor, we used a smoother for time, the study herd and an ordered group factor for juvenile females (n=7) as the base, and adult females (n=7) and juvenile males (n=4) as contrasts. We first fitted three generalized additive mixed models (GAMM). i: Time in an interaction with the demographic variable and the study herd, testing for temporal trends compared to the base group over the summer. ii: The time – demography interaction and the herd as fixed part, and a random intercept and slope per individual to account for individual variation. iii: The time – demography interaction as fixed part, and the random intercept and slope for the individuals and a random intercept for each herd accounting for different variation between the herds. The best model was selected by Chi-square testing on the linear functional relationship estimation by maximum likelihood (fREML) scores. To the model with the lowest fREML scores we added an autoregressive model (AR1) structure based on each ID. The Rho value was based on the autocorrelation factor of lag two.

### 2.10 Body temperature

As for the HR, we fitted a generalized additive mixed model with the daily mean T_b_ as a response variable. Only lambs had abdominal T_b_ sensors, thus sex, but not age, was added as a predictor. The herd was added in an interaction with time (day of the year) and animal ID as random factors. To control for the autocorrelation of the T_b_ measurements we used an AR1 structure based on each ID. The Rho value was based on the autocorrelation factor of lag two and then adjusted based on maximum likelihood values of the same model structure and neighboring Rho values.

### 2.11 Daily variation and circadian rhythm

We estimated the probability of periodicity in the T_b_ and HR data using Lomb Scargle periodograms from the R-package lomb as described in Ruf (1999). For each lamb, raw one-minute t_b_ data from mid-June to end of August was analyzed. The same period applied for the HR data, but we selected quality 0 & 1 and 10 min measurement intervals. Lomb Scargle periodograms are suited for unequally spaced time series (Ruf, 1999). For every day, we selected data 4 days prior and 4 days after (i.e. 9 days) and tested for presence of rhythms between 2 to 30 hours. For each period we selected the highest significant peak, rounded it to the nearest half-hour and calculated the proportion of the detected significant rhythms. For all periods with significant 24-hour rhythms, we selected the daily zenith (max) and nadir (min) T_b_ and HR values from a smoothed data set (rolling mean +/- 2 hours). We report the acrophase as mean time of the day of high and low peak and the amplitude as daily variation.

### 2.12 Growth

Body weight of all lambs was measured at day of birth. All lambs were weighed prior to surgery, and fall weight was taken at the end of the grazing season. Weight gain from birth to fall was compared between lambs with sensors and the control group. We used the restricted maximum likelihood method of the MIXED procedure in SAS™ software, version 9.4. Growth in gram per day was the response, and we added factorial variables for presence/absence of implanted sensor, sex and rearing rank as fixed effects. If more than two lambs are born per ewe, usually the surplus is taken from the ewe and bottle-fed. Rearing rank is a two-digit number where the first digit indicates number of lambs born and the second number of lambs raised by the ewe. Day of birth was included as a numeric fixed effect and the ewe ID as a random effect.

## 3 Results

### 3.1 Sensors found

In total 22 (73 %) of the 30 implanted HR sensors and 32 (80 %) of 40 implanted T_b_ sensors could be retrieved. From the coastal herd, four of 10 HR sensors and 17 of 20 T_b_ sensors could be retrieved (Table 1). Of the 4 HR sensors from the coastal herd, one had a power failure and data collection stopped in late August. From the inland herd 18 of 20 HR sensors and 15 of 20 T_b_ sensors could be retrieved. Of the 18 retrieved HR sensors from the inland herd, four had power failure and data collection stopped late in the study period. All 17 retrieved T_b_ sensors from the coastal herd and all 15 sensors of the inland herd worked as programmed. All lost sensors were either ejected by the sheep or not detected at retrieval as no sheep were missing. From the ten inland ewes eight HR sensors could be retrieved.

**Table 1:** Implanted and retrieved subcutaneous heart rate (HR) and abdominal body temperature (T_b_) sensors.

	Herd	Implanted	Retrieved	Lost
HR sensors	Coastal	10	4	6
	Inland	20	18	2
	Total	30	22	8
T_b_ sensors	Coastal	20	17	3
	Inland	20	15	5
	Total	40	32	8

### 3.2 Clinical examination

During the first 55 days post-surgery, eight lambs from the coastal herd needed treatment because of infected wounds. All eight lambs got a local treatment of antibiotics.

Of eight lambs loosing sensors, five had HR and T_b_ sensors, but only lost the HR sensor, one lost both sensors and two only lost the T_b_ sensor. One lamb without HR sensor lost the T_b_ sensor. Clinical examination was suitable to confirm presence of HR sensors only. At post surgery day 12, one lamb had lost the sensor, another at day 16 and one at day 20. Three lambs lost the sensor between day 32 and 53. One lamb died at day one post surgery, cause of death was a crack in the right heart auricle, which appeared to be increased in size. One more lamb from the coastal herd died during the study period due to coccidiosis (*Eimeria spp.).* From the inland herd one ewe died during the study most likely of tympanitis. At slaughter, two T_b_ sensors were collected from inside the abomasum of two lambs from the coastal herd.

### 3.3 Quality of the HR measurements

Raw ECG data was available for a total of 1720 HR measurements. 130 (7.6%) of those were dominated by noise, had a manual quality assignment of level IV and HR could not be calculated. 1493 (86.8%) measurements were below the suggested quality threshold. In 36 (2.1%) cases with manual quality IV, the on board processing assigned the quality as < 2, detecting a false real from noise. 133 (7.7%) HR measurements had an algorithm measurement quality > 2 but a manual quality < III, leading to deleted measurements of high ECG quality or real false. Following the recommendation and selecting only measurements of high quality (0&1), the quality assignment of the algorithm succeeded in 97.6 % of the cases (Table 2).

**Table 2:** Measurement quality. HR measurements with recommended algorithm quality <2 (0 & 1) related to the manual quality. Manual quality IV: no HR detectable, III: R or S peaks visible, amplitude irregular; noise covers details, II: QRS complex visible; amplitude of the measurement regular; P, T and U wave partly covered by noise, I: QRS complex clearly visible; amplitude of the measurement regular; no or very little noise.

Manual quality	Number measurements (%)
I – IV	1493 (100)
I	631 (42.3)
II	575 (38.5)
III	251 (16.8)
IV	36 (2.4)

The mean difference between the ECG based HR calculated by the algorithm and the HR based on the manually measured time between the first and last QRS interval was 0.20 bpm ranging from -80.85 bpm to 17.76 bpm with a standard deviation of 5.2.

Comparing the sensor-calculated HR to the measured HR, the model with a random intercept for the measuring period and the animal ID had a lower Akaike information criterion (AIC) value (delta > 2) than the model without or either animal ID and measuring period alone. During the stepwise backward selection, first the interaction of age and measured HR was dropped and then the fixed factors, age and period were dropped. The top model remained with the measured HR and the random intercepts for measurement period and animal ID. The added smoother on the measured HR used one degree of freedom and did not show any potential nonlinear relation and was dropped too. Over the full range, the manually measured HR was 1.01 (SE <0.01) bpm higher than the sensor-calculated HR.

The ECG-confirmed minimum HR was 68 bpm measured in an adult female. The ECG-confirmed maximum HR was 197 bpm measured in a juvenile male (Table 3).

**Table 3:** Minimum and maximum ECG based recorded HR in adult and juvenile domestic sheep.

	Min HR bpm	Max HR bpm
Adult females	68	143
Juvenile females	78	190
Juvenile males	82	197

Behavior was recorded for six ewes and five lambs on the mountain pasture of the inland herd for a total of 43 observing hours ranging from 1 hour to 6 hours per individual, resulting in 755 HR measurements. The HR sensor of one ewe was lost and is not included (Table 4).

**Table 4:** Mean HR in bpm for active and passive behaviors in domestic sheep. Adult eves (n=5), juvenile males (n=4) and female (n=1).

	Mean active HR (SD)	Mean passive HR (SD)
Adult eves	106 (17); n=215	90 (13); n=164
Juvenile lambs	128 (18); n=237	112 (13); n=139

### 3.4 Seasonal patterns

**Fig. 1:**
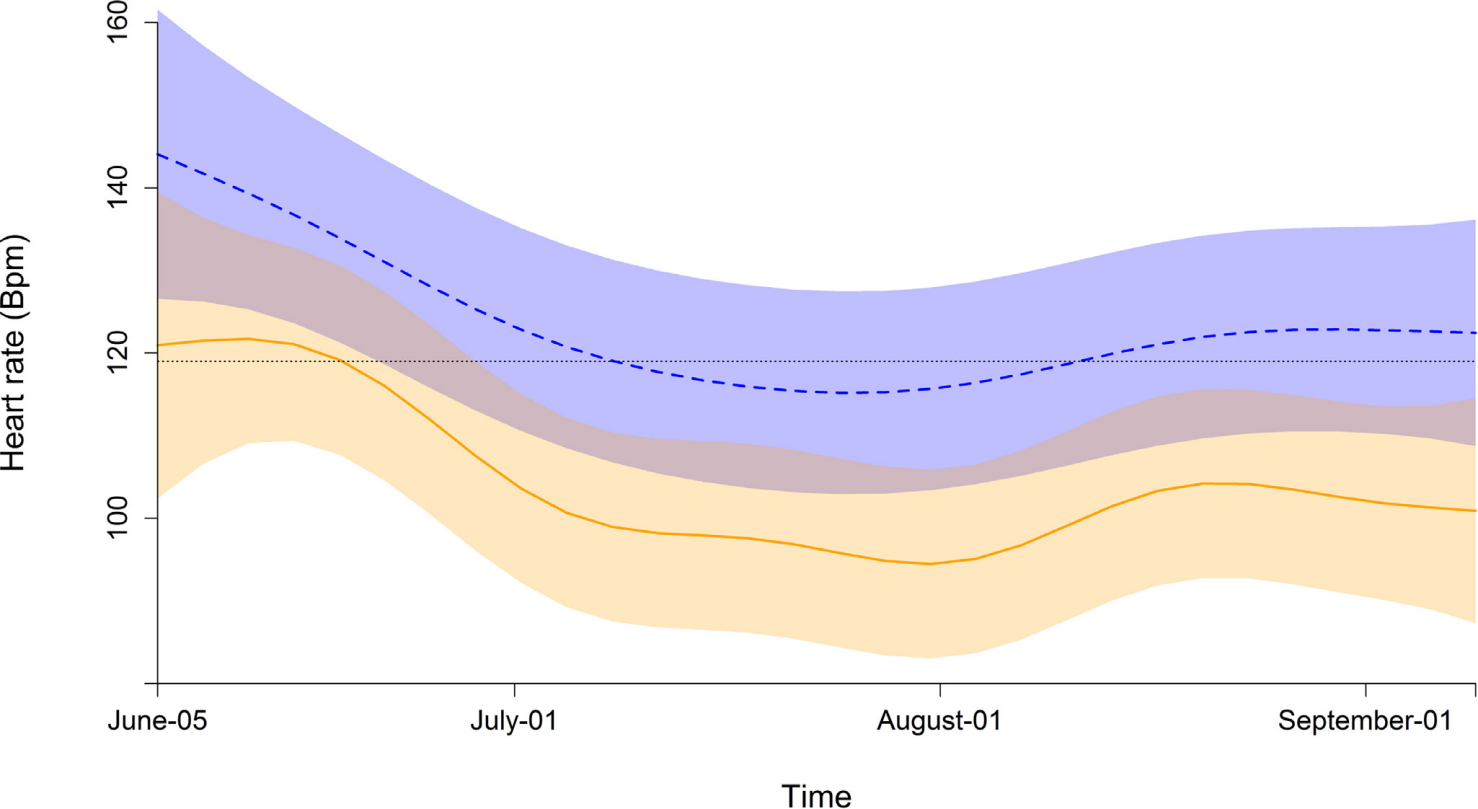
**Fitted mean heart rate (Bpm) and 95% confidence intervals.** Juvenile males (dashed blue line) and adult females (solid orange line) compared to the standardized juvenile females (dotted black line) in an interaction with time. The random terms (herd and ID) are canceled for optical model interpretation.

**Fig. 2:**
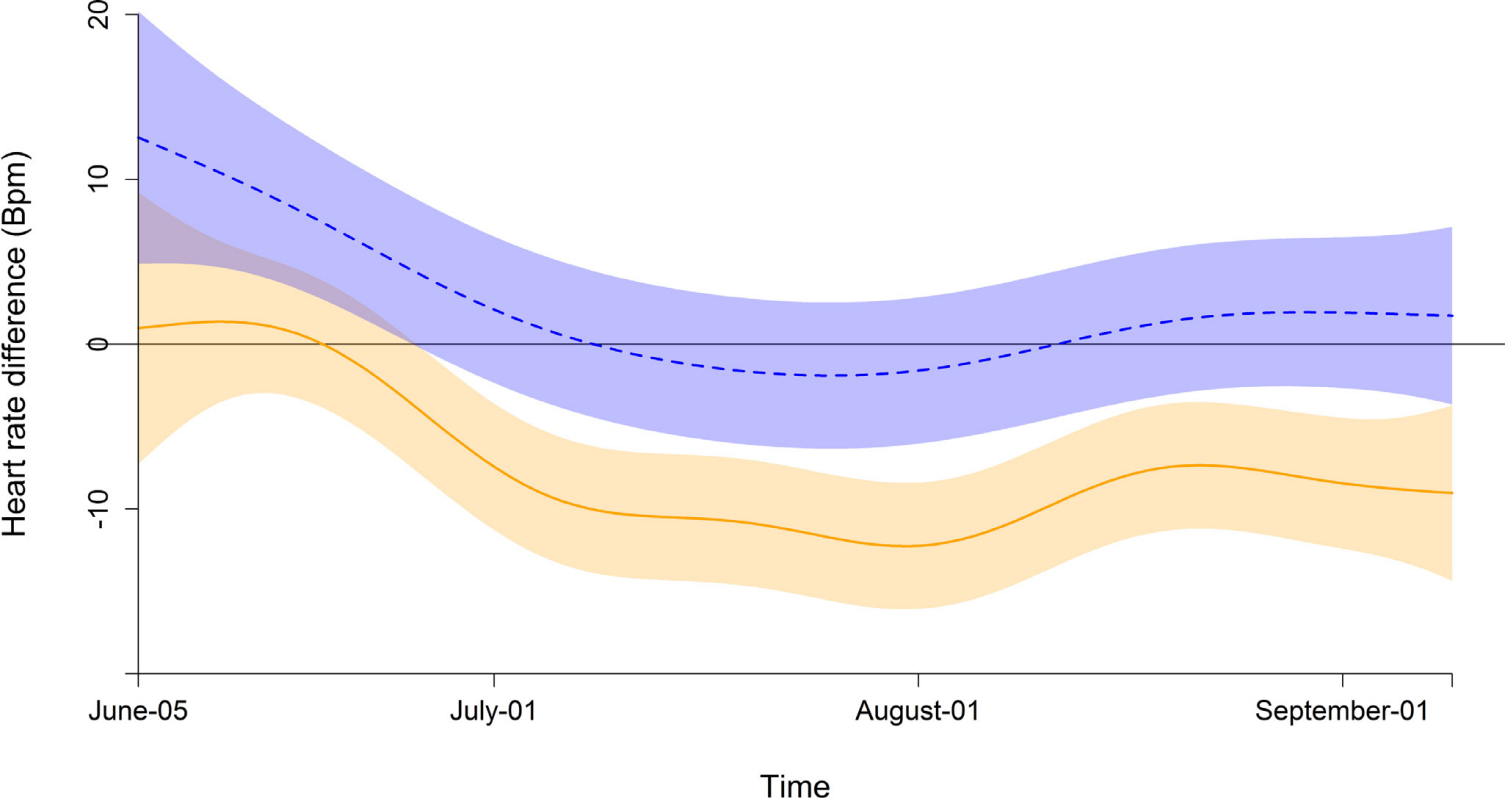
**Estimated difference in HR and 95% confidence intervals between demographic groups.** Difference in HR to juvenile females for juvenile males (dashed blue line) and adult females (solid orange line). No overlap between the confidence interval and the zero line indicates a significant difference.

The model relating HR to the time * demography interaction as fixed part, the random intercept and slope for the individuals and the random intercept for each herd had the lowest fREML scores. The optical residual analysis of the model showed an approximated T-distribution of the residuals but high residual autocorrelation. Adding the AR correlation structure decreased the residual autocorrelation to an acceptable level. Models with AR structure and a Rho value of 0.75 had a lower maximum likelihood as compared to the autocorrelation of lag two (Rho 0.66) and surrounding levels. The interaction smooth of time and demographic group (juvenile females, adult females and juvenile males) did improve the model based on maximum likelihood comparison. All three demographic groups had lower heart rates in July than in June and August (Fig. 1). Compared to the juvenile females the adult females had a different trend over time (estimated DF: 6.0, F = 6.2, p < 0.001) and also the juvenile males differed over time (estimated DF: 3.65, F = 3.9, p = 0.003) (Fig. 2). However the difference between the juvenile males and females was only significant for ~ 19 days in the beginning of the study period, whereas the difference between adult females to juvenile females was lower from end of June through the study period (Fig. 2). This is also reflected in the estimates of the model (Table 5) with no significant difference in the mean HR from juvenile males compared to juvenile females but a lower HR for adult females.

**Table 5:** Estimated HR in bpm for each demographic group. Estimates from the generalized additive mixed model with demographic group as fixed ordered factor and the interaction of the demographic group and time as a smooth term (Fig. 1 and 2) and a random intercept and slope over time for ID and herd as random terms.

	Estimate (SE)	t-value	p-value
juvenile females	119 (5)		
juvenile males	122 (3)	0.90	0.368
adult females	107 (3)	-5.68	< 0.001

**Fig. 3:**
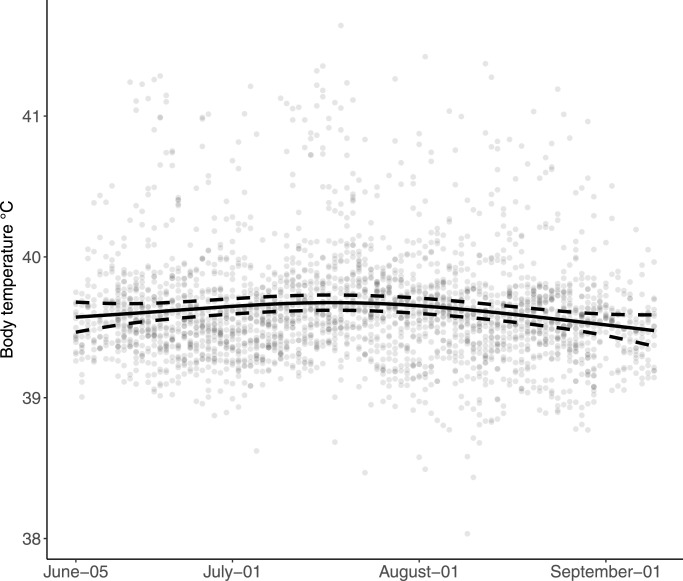
**Body temperature of the lambs from both herds during the study period.** The black solid line shows the fitted values with 95% confidence intervals as dashed lines from the generalized additive mixed model. They grey dots show the daily mean body temperature for all individuals.

Abdominal T_b_ of all lambs in both herds ranged from 36.9°C to 41.8°C with a mean of 39.6°C (SD 0.35). The smooth term in the model was significant (estimated DF: 2.3, F = 3.39, p = 0.02) suggesting a slight seasonal effect on the T_b_ in the second half of July (Fig. 3). The model with sex as predictor was not substantially different to the model without and the term was dropped.

### 3.5 Daily variation and circadian rhythm

In all lambs, T_b_ displayed significant rhythmicity during all the 2250 analyzed periods. We found 24-hour circadian rhythms during 80.7% and 12-hour ultradian rhythms during 9.9% of the periods. The remaining 9.4% were divided to other values with frequencies <5%. From the HR data significant rhythmicity was detected on 78.9% of the 1342 analyzed periods. Significant 24 hour rhythms were detected during 41.0% of the analyzed periods and 15.6% showed a 12-hour ultradian rhythm. The remaining significant periods (22.3%) were divided on other values with frequencies <5% each. Within periods of significant 24h hours circadian rhythm circular mean time of the high peak was at 19:46 GMT (SD = 62min) and low peak at 05:46 GMT (SD = 45min) for T_b_ (local summer time is GMT + 1 hour). HR high peak was earlier at 19:03 GMT (SD = 54min) and low peak at 3:38 GMT (SD = 47min). Smoothed T_b_ ranged from 37.4°C to 42.1°C with an average daily amplitude of 2.0°C (SD = 0.7°C). The average daily span during the 24-hour circadian rhythm in the HR data was 55 bpm (SD = 15 bpm).

### 3.6 Weight gain

Implanting sensors by surgery may affect weight gain of lambs and weight gain in sensor-lambs was therefore compared to weight gain of the remaining lambs in the herd. There were no significant difference in weight gain between lambs with sensors and lambs without sensors within the coastal and inland herd respectively; sensor vs no-sensor lam was 217g vs 222g (p=0.78) in the inland herd and 323g vs 316g (p=0.66) in the coastal herd.

## 4 Discussion

We applied 30 HR and 40 T_b_ sensors in domestic sheep for one summer, tested the accuracy of the HR values and analyzed seasonal and diurnal patterns of HR and T_b_. The HR values reflected the R-R interval measured by the ECG and the sensor successfully filtered bad quality measurements. A major problem was the loss of 27 % of the HR and 20 % of the T_b_ sensors. However, the surgical implantation of the sensors worked without severe complications. The difference between the sensor-calculated HR of the highest quality (QI=0) and the manually calculated HR of one beat per minute was negligible, as in addition this difference was constant over the entire range of the HR measurements.

The HR sensor turns the ECG waveform into a HR in bpm and provides a quality estimate of the measurement. This quality estimate is based on the regularity of the R-R intervals. Large variation between the R-R intervals within a measurement decreases the quality of the saved value. Noise from other muscle contraction generate electromyogram (EMG) signals which can cover parts or entire ECG strips, leading to undetected R peaks and high R-R interval variation. All manually examined ECG strips of low quality were due to such noise. Several other reasons can lead to irregularities of R-R intervals. Pronounced respiratory sinus arrhythmia, as for example known for dogs (Hanton and Rabemampianina, 2006) or hibernating black bears (Laske et al., 2010) can prolong single R-R intervals, a concern for short measurement periods (as in this case 3 sec) in combination with low HR. Some individuals in this study showed arrhythmic R-R intervals for unknown reasons. None of these have been of a major concern for the accuracy of the HR measurements. Selecting for the high quality measurements, this HR sensor provides reliable HR values in domestic sheep. An important factor of success using a HR sensor on a new species is a pilot study. For this study, we used two ewes and adjusted the ECG processing algorithm prior to implantation in the described animals.

Mean HR values in this study (90 to 106 bpm for adult sheep) are within the wide range reported previously. Baldock et al. (1988), the only other study sensing long term HR data on sheep in an outdoor setting, reports lower passive HR of around 70 bpm and similar active HR of 90 bpm to 120 bpm depending on behavior and season for ewes. In two other studies (Arieli et al., 2002; Barkai et al., 2002) focusing on energy expenditure, the authors used, similar to Baldock et al. (1988), external sensors attached with harnesses to the sheep. They report mean HR for lambs from 75 bpm to 102 bpm depending on the time of the day, lower than our lambs with 112 bpm to 128 bpm depending on their activity. Other studies were done with standard ECG for short periods, often in closed pens (Desire et al., 2004) or even restraint (Lopes et al., 2016). Results are presented in mean R-R interval length ranging from 400 ms to 655 ms corresponding to 92 bpm and 150 bpm. This deviation could be due to breed differences. Stubsjøen et al. (2009) recorded mean HR values in adult Norwegian White ewes, the same breed as in this study, in response to mild to moderate ischaemic pain. The ewes received different treatments during a testing period of 60 minutes, and the mean HR varied according to test days and treatments, ranging from 63-73 bpm.

The seasonal pattern with lower HR values in midsummer was unexpected. Previous reports on sheep grazing on fenced grass fields showed increased HR values towards mid-summer (Baldock et al., 1988). Also, wild ruminants, such as red deer *(Cervus elaphus)* (Turbill et al., 2011) or ibex *(Ibex ibex)* (Signer et al., 2011), show distinct seasonal increase in HR towards mid-summer. In a review, Brosh (2007) pointed out the positive effect of feed quality and schedule as well as locomotor activity on HR and energy expenditure. On range pasture, our study animals choose their schedule free. Feed quality within the range pasture is highly variable and we have no knowledge on how the different studied ewes selected feeding grounds. Assumingly they search a tradeoff between feed quality, environmental conditions and competition. The increased HR appears when the sheep were on fertilized clover/grass pasture prior and post grazing season. The nutritional quality as well as the physiological stress and supplementation with concentrated feed following regular handling could together explain some of the increase in HR. However Turbill et al. (2011) shows a limited effect of nutrition intake on the resting HR of red deer compared to the seasonal effect and the here observed seasonal effect has to be interpreted with care.

The presence of 24-hour circadian rhythms based on T_b_ and HR confirms earlier studies on activity-based biological rhythms studied in sheep (Nunes Marsiglio Sarout et al., 2018; Warren and Mysterud, 1991; Wyse et al., 2018). All three studies mention disruption of circadian rhythms by environmental conditions and Wyse et al. (2018) shows environmentally induced immediate and profound changes. The steady presence of circadian rhythm based on T_b_ in our study, but less apparent rhythmicity based on HR, might reflect a similar pattern, leading to the assumption that Tb represents a robust basal rhythmicity whereas HR rather reflects behavioral changes. The combination of T_b_ and HR with different variations in their rhythmicity could have high potential for early disease detection. The delayed daily peaks of T_b_ compared to HR are similar to the patterns found in ibex during summer (Signer et al., 2011) and in accordance with the relation of HR and heat production (Brosh, 2007).

A higher proportion of T_b_ sensors could be retrieved from the coastal herd as compared to the inland herd. The lambs from the coastal herd were slaughtered in a small facility and all internal organs could be searched independently of the slaughter line. We are confident that all sensors, both HR and T_b_, that were not found in the carcass have been ejected by the lambs during the summer. However, in the inland herd, the retrieval of the sensors took place in a large industrial slaughter facility with little flexibility or time to search the carcasses properly. Consequently, we assume some sensors may have been lost during slaughtering. Still there was a lower proportion of retrieved HR sensors from the coastal herd, very likely due to a higher loss of sensors during grazing season. The preventative medication of the inland herd at surgery probably increased healing quality. However the late loss of 3 sensors (between 32 and 53 days post-surgery) is unlikely due to direct infection from the surgery. Lambs in the coastal herd had likely a 100 % prevalence of *Anaplasma phagocytophilum* (*A.ph*.) infection that may cause TBF (Grøva et al., 2013). TBF decreases the bactericidal activity of neutrophils increasing the risk of infections (Woldehiwet, 2010). Neutrophils are also the first cell type present in the inflammatory response and dominating the early exposure to a implanted biomaterial (Anderson and Cramer, 2015). Increased susceptibility to infections also increases the risk of bacterial engraftment on biomaterials, which may lead to abscess formation and sepsis. (Londono and Badylak, 2015). The two T_b_ sensors retrieved from the digestive system might have been on the way out, suggesting a potential path for ejection from the abdominal cavity.

The surgical implantation, although only taking 6-13 min, is not applicable on low value livestock animals or even in general for an agricultural setting. Alternatives are needed, and possibly in the form of a rumen bolus sending T_b_ to a neck collar, or an insertable subcutaneous temperature sensor in the form of a microchip. HR could be measured by light technology, similar to HR watches for humans, perhaps directly from the neck collar. An additional challenge is the sensor-to-farmer communication. For free grazing production animals, a combination of spatial and physiological data could have high value for individual and herd monitoring. Physiological data from the sensors may be sent to a GPS neck collar communicating by GSM network or satellite to the farmer, delivering information about the health status and the position of the individuals in the herd. Currently, a number of commercial position tracking collars are available i.e. Telespor and FindMySheep. Also, a number of external sensor collar systems that communicate are available for indoor or small-scale pasture settings e.g. (Zehner et al., 2017) or in research settings followed by high costs (Signer et al., 2011).

With this study we lay out a baseline for physiological values of HR and T_b_ for sheep on rangeland summer pastures and tested current sensor technology available. To form a PLF system, three steps need to be accomplished: *i* Communication between sensors and the animal transceiver hub (neck collar). *ii* Communication of sensor information from the animal to the farmer and *iii* automated data analysis for an early warning system to the farmer.

## 5 Availability of data and materials

Demographic variables of the sheep, heart rate and body temperature data is available as used in the analysis upon request to the corresponding author.

## Declaration of Competing Interest

All authors declare no conflict of interest associated with this article.

## Appendix

### Surgical technique

All surgeries were conducted inside the barn. On a clean table, we prepared medicines, equipment and sterilized surgical kits for each animal. The surgeon and three assistants handled the sheep during the surgery, while a second team prepared the next animal for surgery. All sheep were moved from the pen immediately before preparing and put back to the pen after the surgery. Prior to surgery, all sheep were weighed, rectal temperature was measured and for each surgery a protocol was written. All sensors were sterilized using a 12-hour gas sterilizer and propylene gas.

### Subcutaneous sensor

The ewes were placed in a position similar to when they are sheared, i.e. sitting on their back, with one person holding firmly. Lambs were placed in dorsal recumbency on a V-form bench. The sensor (Milli HRT) was placed subcutaneous on the left thorax, exactly above the heart and with the sensor axis parallel to the heart axis. This position was identified by auscultation of the heart, and marked with a pen on the skin. The skin was prepared for surgery, and local anesthesia Lidokel-Adrenalin vet. Kela ® (ATCvet-nr.: QN01B B52), 20 mg/ml, 1 - 2 ml was administered subcutaneously. All lambs got 0.5 ml Metacam vet. Boehringer Ingelheim Vetmedica GmbH (ATCvet-nr.: QM01A C06), i.m. as an analgesic. After minimal five minutes, a skin incision of 2 cm was made 90 degrees on the selected axis for the sensor. The skin (epidermis and subcutis) was loosened from the thorax using forceps creating a pocket to insert the sensor with the two electrodes facing to the skin. The skin was sutured by 2-3 sutures of 2-0 Prolene, and the scar was sprayed with liquid plaster. The procedure, including the anesthesia, took approximately 12 min.

### Abdominal sensor

The abdominal temperature sensor (centi-T) was placed in the abdomen near by the umbilical cord. The skin was shaved and prepared and local anesthesia –1 ml Lidokel-Adrenalin vet. Kela ® (ATCvet-nr.: QN01B B52), 20 mg/ml, was given. The Lidokain was distributed to the epidermis, subcutis and the abdominal muscles. After preparation, the lambs were allowed to rest for 10-15 minutes for the analgesic to take effect before the surgery started. An incision of about 2 cm through the skin and the abdominal muscles and peritoneum was made, and the sterilized sensor was put into the abdomen. The sensor was sutured to the abdominal wall using Prolene 2-0 suture. The linea alba was sutured with a continuous suture interrupted with one knot midway. The skin (epidermis and sub cutis) was closed with 2-3 interrupted matrass sutures of Prolene 2-0. The incision was treated with 0.2 ml penicillin (Penovet vet. Boehringer Ingelheim Danmark A/S (ATCvet-nr.: QJ01C E09) 300 mg/ml) and liquid plaster.

In the inland herd, all the lambs got Terramycin prolongatum inj, 0.5 ml i.m. This was based on experience from the coastal herd where we had some cases of infected wounds after the surgery. The surgical site will not be sterile when operating in a barn and the lambs will be exposed to infections in the operation scars the first 2-3 days.

The procedure from incision to closing and the lamb was back in the pen, took between 6 and 13 minutes.

## References

[bib0001] Anderson J., Cramer S., Badylak S.F. (2015). Perspectives on the Inflammatory, Healing, and Foreign Body Responses to Biomaterials and Medical Devices. Host Response to Biomaterials.

[bib0002] Andersson L.M., Okada H., Miura R., Zhang Y., Yoshioka K., Aso H., Itoh T. (2016). Wearable wireless estrus detection sensor for cows. Comput Electron Agric.

[bib0003] Arieli A., Kalouti A., Aharoni Y., Brosh A. (2002). Assessment of energy expenditure by daily heart rate measurement—validation with energy accretion in sheep. Livestock Production Science.

[bib0004] Baldock N.M., Sibly R.M. (1990). Effects of Handling and Transportation on the Heart-Rate and Behavior of Sheep. Appl Anim Behav Sci.

[bib0005] Baldock N.M., Sibly R.M., Penning P.D. (1988). Behavior and Seasonal-Variation in Heart-Rate in Domestic Sheep, Ovis-Aries. Anim Behav.

[bib0006] Barkai D., Landau S., Brosh A., Baram H., Molle G. (2002). Estimation of energy intake from heart rate and energy expenditure in sheep under confinement or grazing condition. Livestock Production Science.

[bib0007] Bates D., Machler M., Bolker B.M., Walker S.C. (2015). Fitting Linear Mixed-Effects Models Using lme4. J stat soft.

[bib1045] Bjarnason Á., Gunnarsson A., Árnason T., Oddgeirsson M., Sigmarsson A.B., Gunnarsson Á. (2019). Validation of ECG-derived heart rate recordings in Atlantic cod (Gadus morhua L.) with an implantable data logging system. Anim Biotelemetry.

[bib0008] Brosh A. (2007). Heart rate measurements as an index of energy expenditure and energy balance in ruminants: A review. J Anim Sci.

[bib0009] De Wet L., Vranken E., Chedad A., Aerts J.M., Ceunen J., Berckmans D. (2003). Computer-assisted image analysis to quantify daily growth rates of broiler chickens. Br Poult Sci.

[bib0010] Desire L., Veissier I., Despres G., Boissy A. (2004). On the way to assess emotions in animals: Do lambs (Ovis aries) evaluate an event through its suddenness, novelty, or unpredictability?. J Comp Psychol.

[bib1018] Dwyer C. (2008). The Welfare of Sheep.

[bib0011] Evans A.L., Singh N.J., Fuchs B., Blanc S., Friebe A., Laske T.G., Frobert O., Swenson J.E., Arnemo J.M. (2016). Physiological reactions to capture in hibernating brown bears. Conserv Physiol.

[bib0012] Ewald P.W. (1980). Evolutionary biology and the treatment of signs and symptoms of infectious disease. J Theor Biol.

[bib0013] Fehlmann G., King A.J. (2016). Bio-logging. Curr Biol.

[bib0014] Forin-Wiart M.-A., Enstipp M., Le Maho Y., Handrich Y. (2018). Why implantation of bio-loggers may improve our understanding of how animals cope within their natural environment. Integr Zool.

[bib0015] Friebe A., Evans A.L., Arnemo J.M., Blanc S., Brunberg S., Fleissner G., Swenson J.E., Zedrosser A. (2014). Factors affecting date of implantation, parturition, and den entry estimated from activity and body temperature in free-ranging brown bears. PLoS ONE.

[bib0016] Green J.A. (2011). The heart rate method for estimating metabolic rate: review and recommendations. Comp Biochem Physiol A Mol Integr Physiol.

[bib0017] Grøva L., Olesen I., Steinshamn H., Stuen S. (2013). The effect of lamb age to a natural Anaplasma phagocytophilum infection. Small Ruminant Res.

[bib0018] Guarino M., Jans P., Costa A., Aerts J.M., Berckmans D. (2008). Field test of algorithm for automatic cough detection in pig houses. Comput Electron Agric.

[bib0019] Hanton G., Rabemampianina Y. (2006). The electrocardiogram of the Beagle dog: reference values and effect of sex, genetic strain, body position and heart rate. Lab Anim.

[bib0020] Hume D.A., Whitelaw C.B.A., Archibald A.L. (2011). The future of animal production: improving productivity and sustainability. J Agric Sci.

[bib0021] Jore S., Viljugrein H., Hofshagen M., Brun-Hansen H., Kristoffersen A.B., Nygard K., Brun E., Ottesen P., Saevik B.K., Ytrehus B. (2011). Multi-source analysis reveals latitudinal and altitudinal shifts in range of Ixodes ricinus at its northern distribution limit. Parasit Vectors.

[bib0022] Kilkenny C., Browne W.J., Cuthill I.C., Emerson M., Altman D.G. (2010). Improving bioscience research reporting: the ARRIVE guidelines for reporting animal research. PLoS Biol.

[bib0023] Laske T.G., Harlow H.J., Garshelis D.L., Iaizzo P.A. (2010). Extreme respiratory sinus arrhythmia enables overwintering black bear survival–physiological insights and applications to human medicine. J Cardiovasc Transl Res.

[bib0024] Londono R., Badylak S.F., Badylak S.F. (2015). Chapter 1 - Factors Which Affect the Host Response to Biomaterials. Host Response to Biomaterials.

[bib0025] Lopes D.I., Sousa M.G., Ramos A.T., Maruo V.M. (2016). Cardiotoxicity of Senna occidentalis in sheep (Ovis aries). Open Vet J.

[bib0026] Marais M., Maloney S.K., Gray D.A. (2013). Sickness behaviours in ducks include anorexia but not lethargy. Appl Anim Behav Sci.

[bib0027] Mustafa M. (2015). Ownership Identification of Reindeer Calf Using Wireless Sensor Networks (WSN). Communications.

[bib0028] NIBIO, 2017a. National statistics on grazing animals on county level 1970 - 2016. (url:https://www.nibio.no/tema/landskap/kart-over-beitebruk-og-seterdrift/beitestatistikk?locationfilter=true), access date: 31. May 2018.

[bib0029] NIBIO, 2017b. National statistics on grazing animals on grazing area level 2013-2016. (url:https://www.nibio.no/tema/landskap/kart-over-beitebruk-og-seterdrift/beitestatistikk?locationfilter=true), access date: 31. May 2018.

[bib0030] Nkrumah J.D., Okine E.K., Mathison G.W., Schmid K., Li C., Basarab J.A., Price M.A., Wang Z., Moore S.S. (2006). Relationships of feedlot feed efficiency, performance, and feeding behavior with metabolic rate, methane production, and energy partitioning in beef cattle1. J Anim Sci.

[bib0031] NRK, 2017. Norwegian Meterological institutt and NRK. (url:https://www.yr.no/sted/Norge/Hedmark/Tynset/Tynset/statistikk.html), access date: 14.04.2017.

[bib0032] Nunes Marsiglio Sarout B., Waterhouse A., Duthie C.-A., Candal Poli C.H.E., Haskell M.J., Berger A., Umstatter C. (2018). Assessment of circadian rhythm of activity combined with random regression model as a novel approach to monitoring sheep in an extensive system. Appl Anim Behav Sci.

[bib0033] Piccione G., Caola G., Refinetti R. (2002). Effect of shearing on the core body temperature of three breeds of Mediterranean sheep. Small Ruminant Res.

[bib0034] Core Team R (2014). R: A language and environment for statistical computing. Version: 3.0.3.

[bib0035] Reganold J.P., Wachter J.M. (2016). Organic agriculture in the twenty-first century. Nat Plants.

[bib0036] Rovbase, 2018. National statistics on predation and compensation of predation on sheep and reindeer. (url:www.rovbase.no), access date: 31 May 2018.

[bib0037] Ruf T. (1999). The Lomb-Scargle Periodogram in Biological Rhythm Research: Analysis of Incomplete and Unequally Spaced Time-Series. Biol Rhythm Res.

[bib0038] Signer C., Ruf T., Arnold W. (2011). Hypometabolism and basking: the strategies of Alpine ibex to endure harsh over-wintering conditions. Funct Ecol.

[bib0039] Stubsjøen S.M., Flo A.S., Moe R.O., Janczak A.M., Skjerve E., Valle P.S., Zanella A.J. (2009). Exploring non-invasive methods to assess pain in sheep. Physiol Behav.

[bib2045] Thiel A., Evans A.L., Fuchs B., Arnemo J.M., Aronsson M., Persson J. (2019). Effects of reproduction and environmental factors on body temperature and activity patterns of wolverines. Front Zool.

[bib0040] Trethowan P.D., Hart T., Loveridge A.J., Haw A., Fuller A., Macdonald D.W. (2016). Improved homeothermy and hypothermia in African lions during gestation. Biol Lett.

[bib0041] Turbill C., Ruf T., Mang T., Arnold W. (2011). Regulation of heart rate and rumen temperature in red deer: effects of season and food intake. The Journal of Experimental Biology.

[bib0042] Van Hertem T., Rooijakkers L., Berckmans D., Peña Fernández A., Norton T., Berckmans D., Vranken E. (2017). Appropriate data visualisation is key to Precision Livestock Farming acceptance. Comput Electron Agric.

[bib0043] van Rij, J., Wieling, M., Baayen, R., & van Rij, H. (2016). itsadug: Interpreting Time Series and Autocorrleated Data using GAMMs 2.3. R package.

[bib0044] Verbeke W., Viaene J. (1999). Beliefs, attitude and behaviour towards fresh meat consumption in Belgium: empirical evidence from a consumer survey. Food Quality and Preference.

[bib0045] Warren J.T., Mysterud I. (1991). Summer Habitat Use and Activity Patterns of Domestic Sheep on Coniferous Forest Range in Southern Norway. J Range Manage.

[bib0046] Wikelski M., Cooke S.J. (2006). Conservation physiology. Trends Ecol Evol.

[bib0047] Woldehiwet Z. (2010). The natural history of Anaplasma phagocytophilum. Vet Parasitol.

[bib0048] Wood S.N. (2011). Fast stable restricted maximum likelihood and marginal likelihood estimation of semiparametric generalized linear models. J R Stat Soc B.

[bib0049] Wyse C.A., Zhang X., McLaughlin M., Biello S.M., Hough D., Bellingham M., Curtis A.M., Robinson J.E., Evans N.P. (2018). Circadian rhythms of melatonin and behaviour in juvenile sheep in field conditions: Effects of photoperiod, environment and weaning. Physiol Behav.

[bib0050] Zehner N., Umstatter C., Niederhauser J.J., Schick M. (2017). System specification and validation of a noseband pressure sensor for measurement of ruminating and eating behavior in stable-fed cows. Comput Electron Agric.

[bib0051] Zuur A., Ieno E.N., Walker N., Saveliev A.A., Smith G.M. (2009). Mixed effects models and extensions in ecology with R.

